# The prevalence of hearing loss in an elderly population in Rio de Janeiro: a cross-sectional study

**DOI:** 10.1016/S1808-8694(15)30126-9

**Published:** 2015-10-19

**Authors:** Leila Couto Mattos, Renato Peixoto Veras

**Affiliations:** a Doctor, Audiologist; b Doctoral thesis, Director of the Open University for Elderly Persons, UnATI/UERJ; professor at UERJ; Researcher level 1, CNPQ. Rio de Janeiro State University - UERJ

**Keywords:** aging, epidemiology, hearing loss, presbyacusis, prevalence

## Abstract

Hearing loss in the elderly is one of the most frequent chronic diseases. **Aim:** to estimate the prevalence of hearing loss in a representative sample of elderly people (aged 65 or over) in the city of Rio de Janeiro. **Material and Method:** a prospective, cross-sectional population-based study with 238 elderly people aged 65 years or more (198 women and 40 men). **Results:** the prevalence of hearing loss was 39.4% (better ear) and 61.6% (worse ear) in the female group, 60% (better ear) and 77.5% (worse ear) in the male group, and 42.9% and 64.3% in the study population. Mild hearing loss was the most prevalent level of hearing loss. **Conclusion:** the prevalence of hearing loss in the study population was significant and in accordance with others relevant international epidemiological studies. Longitudinal studies are needed to better understand age-related hearing loss.

## INTRODUCTION

A number of papers have noted the age-related decline of hearing sensitivity in humans;^1^, ^2^, ^3^, ^4^, ^5^, ^6^, ^7^, ^8^, ^9^, ^10^, ^11^ hearing loss in fact is one of the most common chronic diseases in the elderly. Epidemiological studies based on standard audiological methods such as pure tone audiometry12 have not been widely conducted in countries such as Brazil, where the elderly population grows rapidly.

Various authors have discussed the consequences of hearing loss in the elderly.^13^, ^14^, ^15^, ^16^ According to Ringdahl,^17^ it appears to be a condition that places these individuals in a risk group for psychosomatic diseases.

Many authors^18^, ^10^, ^19^ have considered hearing loss related to age (presbyacusis) as a multiple-etiology result of various negative extrinsic and intrinsic factors. As a polycausal chronic disease it is difficult to define hearing loss in the elderly as a decline in auditory sensitivity caused only by age-related degeneration.^6^, ^20^ Rosenhall^18^ has suggested the term age-related hearing loss as an alternative expression for presbyacusis, due to a general difficulty in audiological research and practice of defining hearing loss as purely the consequence of natural aging (which could be named pure presbyacusis).

Brazil has witnessed a significant growth of its elderly population. Currently, there are 16,8 million people aged 60 years or more in the country, in a total population of 183 million people.21 It is expected that this number will grow to 32 million by 2024. Brazil is thus included in the world scenario of increased human longevity, which has reached previously unthought-of limits.^22^

According to the national health policy for physically challenged persons^23^ presbyacusis is age-related hearing loss, and the main cause of hearing loss in adults. Its estimated prevalence is about 30% in the elderly population, which is defined as people aged 65 or above. Noise, particularly in the working environment, could be defined as the second cause of hearing loss in adults.

It is important to know the prevalence of hearing loss in the elderly, as this information can support specific policies and public services for this age group. According to the national health policy for physically challenged persons,^23^ knowledge of the prevalence of potentially incapacitating diseases and deficiencies becomes an essential basis for developing prevention measures and for adopting strategies aimed at reducing or eliminating the causes of those deficiencies. Early diagnosis, the indication and adaptation of an individual hearing aid, and a program for specific auditory reeducation aimed at the elderly population, become essential issues for better quality of life, and improved family and social integration for the elderly.

This paper aimed to estimate the prevalence of hearing loss in an elderly population in the city of Rio de Janeiro, in Brazil, based on auditory thresholds measured by standard pure tone audiometry.^12^

## STUDY DESIGN

A cross-sectional population-based study was made in 2004 of elderly people aged 65 years or above, registered in an open university for elderly persons in the city of Rio de Janeiro. The sample was composed of 238 subjects selected randomly from the office registry.

## SAMPLE

The study sample was recruited in an open university where the entrance requirement is to be aged 60 years or over. Various courses are offered in this project for elderly persons, who are autonomous and independent people.

The baseline sample included subjects that had registered in the institution between 1993 and 2004; the approximate total number was 3,500 people. The sample population included subjects aged 65 or above that had registered in 2004.

The sample was composed of 258 people (218 women and 40 men) who met the inclusion criteria. Talks were given in the university facilities about age-related hearing loss to guide and inform the subjects about the ongoing study before recruiting of previously selected individuals. Subjects were invited to participate in person, during the talks and by telephone. The talks were given after institutional approval had been obtained.

There were 238 subjects that decided to participate in this study out of the total sample, which was composed of all of the 40 men and 198 women (92.2% of the total sample). The 20 women that did not participate were invited by telephone; six of them had moved to other cities, one had a diagnosis of dementia, two had been diagnosed as depressed, and 11 did not participate for personal reasons.

The state of Rio de Janeiro has the highest rate of elderly persons in Brazil (12.8%), of which most are women. On average women live eight years longer than men.^21^, ^24^ This may explain the unequal gender distribution in our sample. Our sample also contained many women that were widows (45.5%); the men, on the other hand, were mostly married (65%), reinforcing the predominance of women in the sample.^25^

Great care was taken when comparing genders in our sample, given the numeric difference between men and women.

We made a sample calculation to assess the power of our sample based on national health policy estimates for physically challenged persons; these estimates suggest a 30% prevalence of hearing loss in the Brazilian elderly population. We used a 95% confidence level and a 6% acceptable error level. These calculations allowed us to establish that 211 subjects would represent the baseline population.

The research project was sent to the Research Ethics Committee (CEP08/2004) of the Social Medicine Institute/UERJ and of the institution that was responsible for the Open University project, and was approved. All of the participants signed a free informed consent form.

## METHOD

The audiological assessment was made by the audiology team of the Instituto Nacional de Educação de Surdos (INES). INES is a federal public agency that belongs to the Education Ministry; it is a national center for research and study and a school for about 500 deaf students. Pure tone audiometry was part of a detailed evaluation of auditory function in the elderly subjects. Part of the assessment clinical history taking that included information about the health and social history of each patient, and meatoscopy.

### Pure Tone Audiometry

We used the Interacoustic-AD27 for pure tone audiometry calibrated according to ISO/DIS standards.26 The tone was presented separately to each ear through TDH-39 earphones in an appropriate acoustical environment. We used the ascending method to assess airway auditory thresholds at 0.25, 0.5, 1, 2, 3, 4, 6, and 8 kHz. Bone transmission was measured at 0.5, 1, 2, 3, and 4 kHz. Masking was used when necessary. The procedures were conducted according to ISO 8253-1 standards.12 Pure tone averages (PTAs) were calculated at 0.5, 1, 2, and 4 kHz, according to the Editorial Guidelines for the description of inherited hearing loss.27 The ear with the best PTA was considered the better ear, and the worse PTA defined the worse ear. The frequency tone threshold where subjects presented no specific sensitivity was defined at 120 dB HL.

### Hearing Loss

The World Health Organization (WHO)^28^ classification was used to define hearing loss in this study, as shown on Table 1.

**Table 1:** Classification of hearing loss28

PTA (0.5 – 4 kHz)	Verbal descriptor
≤ 25 dB HL	Normal
26 – 40 dB HL	mild
41- 60 dB HL	Moderate
61 – 80 dB HL	Severe
≥ 81 dB HL	Profound

Bilateral hearing loss was present when the PTA was above 25 dB HL in both ears. Unilateral hearing loss was present when the PTA in one ear was equal to or below 25dB dB HL and above 25dB HL in the other ear, or when there was a value over 50dB HL in at least one frequency. Asymmetric hearing loss was present when there was a more than 10 dB HL difference between ears in at least two frequencies, where the PTA in the best ear was equal to or below 25 dB HL. Symmetrical hearing loss was defined when all of the abovementioned options were excluded. These criteria were defined according to the Editorial Guidelines for description of inherited hearing loss.^27^

Mixed hearing loss was defined in audiometry when the bone conduction PTA was above or equal to 25dB HL and a GAP/PTA air-bone equal to or over 15 dB HL. Conduction hearing loss was defined in audiometry when the bone conduction PTA was below or equal to 25 dB HL and a GAP/PTA air-bone equal to or over 15 dB HL. In both cases subjects were referred to the ENT specialist. Subjects with cerumen obstruction returned for the assessment after having the obstruction removed.

## RESULTS

Table 2 shows the distribution of various degrees of hearing loss in women, men and the total sample population distributed according to the best and worse ears.

**Table 2:** Distribution of normal hearing and hearing loss in women (M65-84), men (H65-81) and for the complete sample population (PE65-84) for the best and worse ear (%).

Age; n	Hearing Loss	
	Best ear	Worst ear
M65-84 n=198	78 (39.4)	122 (61.6)
H65 -81 n=40	24 (60)	31 (77.5)
Population n the study (65-84) n= 238	102 (42.9)	153 (64.3)

Table 3 shows the distribution of bilateral and unilateral hearing loss and whether it symmetrical or asymmetrical, in women, men and the total sample population.

**Table 3:** Distribution of bilateral and unilateral, and symmetric and asymmetric hearing loss, for women (M65-84), men (H65-81) and for the complete sample population (PE65-84) (%).

Age; n	Hearing Loss		
	Bilateral		Unilateral
	Symmetrical	Asymmetrical	
M65-84 N=198	45 (22.7)	35 (17.7)	42 (21.2)
H65 -81 n=40	10 (25)	14 (35)	7 (17.5)
Population in the Study (65-84) n= 238	55 (23.1)	49 (20.6)	42 (17.6)

The group of elderly women was composed of 198 women aged between 65 and 84 years (mean: 71.7 years) and the group of elderly men was composed of 40 men aged between 65 and 81 years (mean: 71.9 years). The sample population contained 238 subjects aged between 65 and 84 years (mean: 71.8 years).

In the women’s group (M65-84), 39.4% had some degree of hearing loss in the best ear; 61.6% had some degree of hearing loss in the worst ear. Unilateral hearing loss was found in 21.2% of women; symmetrical hearing loss was found in 22.7% of women and bilateral asymmetric hearing loss was found in 17.7% of women.

In the men’s group (H65-81), 60% had some degree of hearing loss in the best ear; 77.5% had some degree of hearing loss in the worst ear. Unilateral hearing loss was found in 17.5% of men; symmetrical hearing loss was found in 25% of men and bilateral asymmetric hearing loss was found in 35% of men.

In our total sample, 42.9% had some degree of hearing loss in the best ear; 64.3% had some degree of hearing loss in the worst ear. Unilateral hearing loss was found in 17.6% of elderly subjects; bilateral symmetrical hearing loss was found in 23.1% of elderly subjects and bilateral asymmetric hearing loss was found in 20.6% of elderly subjects.

Table 4 shows the distribution of various degrees of hearing loss^28^ for both genders and the total sample, for the best and the worst ear. Mild hearing loss was more prevalent in all of the groups.

**Table 4:** Classification of hearing loss28 for women (M65-84), men (H65-81) and for the complete sample population (PE65-84) for the best and worse ear (%).

Age; n	Hearing loss level	Better ear	Worse ear
M65-84 n=198	≤ 25 dB HL	120 (60.7)	76 (38.4)
	26 – 40 dB HL	52 (26.3)	74 (37.4)
	41 - 60 dB HL	22 (11.1)	34 (17.2)
	61 – 80 dB HL	3 (1.5)	6 (3)
	≥ 81 dB HL	1 (0.5)	8 (4)
H65-81 n=80	≤ 25 dB HL	16 (40)	9 (22.5)
	26 – 40 dB HL	19 (47.5)	17 (42.5)
	41 - 60 dB HL	5 (12.5)	10 (25)
	61 – 80 dB HL	0	1 (2.5)
	≥ 81 dB HL	0	3 (7.5)
Study Population (65-84) n=238	≤ 25 dB HL	136 (57.1)	85 (35.8)
	26 – 40 dB HL	71 (29.9)	91 (38.2)
	41 - 60 dB HL	27 (11.3)	44 (18.5)
	61 – 80 dB HL	3 (1.3)	7 (2.9)
	≥ 81 dB HL	1 (0.4)	11 (4.6)

## DISCUSSION

Our results are similar to those in other epidemiological studies,^10^, ^29^, ^30^ and may be compared according to definition and prevalence of hearing loss criteria in the elderly. Rosenhall18 confirms the results of population-based studies that have shown a higher prevalence of hearing loss in men. Similar results were found in the current study, notwithstanding the small sample size for men (prevalence of hearing loss in women: 39.4%; and in men: 60%).

Two investigations on hearing have been made based on the epidemiological Framingham Heart Study,^31^ one by Moscicki et al.^32^ in 1985 and the other by Gates et al.^10^ in 1990. The latter compiled the data of both trials and concluded that 41% of subjects whose mean age was 73 years presented hearing loss according to common criteria in both studies. These data are close to our findings (42.9% prevalence), based on the best ear - as were the abovementioned trials - for our total sample, where the mean age was 71.8 years.

The results of a paper by Davis A.29 in the United Kingdom revealed a 60% prevalence of hearing loss for the best ear in subjects whose mean was age 75.5 years. Our findings show a 42.9% prevalence in the best ear for a mean age of 71.8 years. The results are relatively similar when we consider the age difference.

Cruickshanks et al.^30^ looked at PTAs in the worst ear at four frequencies in an epidemiological study of subjects whose mean age was 65.8 years, in Wisconsin, USA. He found a 45.9% prevalence of hearing loss, with a higher percentage of mild hearing loss. These findings are similar to our results considering that the mean age in our sample was 71.9 years, and that the prevalence for the worst ear was 64.3%.

The Swedish Gerontological and Geriatric population Study of Göteborg^33^, ^34^ included cross-sectional and longitudinal studies of four cohorts, showing that hearing loss in subjects aged between 70 and 80 years occurred at a rate of 1-2 dB HL/year. Hearing loss progresses at a slower rate between ages 80 and 90 years.

The 30% prevalence of hearing loss among people aged 65 years or more that is stated by the national health policy for physically challenged persons^23^ appears to be outdated according to the latest published findings and to the current study. Even if we take into account the best ear, the prevalence of hearing loss was 39.4%, 60% and 42.9% in each group. These numbers increase to 61.6%, 77.5% and 64.3% for the worst ear.

There was a higher percentage of subjects with mild hearing loss, a finding in common with other international papers.^7^, ^35^, ^13^

The symmetrical type was the most frequent form of hearing loss, which suggests that hearing loss was related to age. There was, however, a higher rate of asymmetrical hearing loss in the male group compared to the female group; this could be explained by a greater exposure of men to noise at work and in leisure activities, to cardiovascular conditions, to smoking, and to other factors such as alcohol abuse, which have also been mentioned in papers relating hearing loss, gender and aging. Our sample, however, is relatively small, which does not allow us to generalize our findings.

## CONCLUSION

The prevalence of hearing loss in our sample was high. There is, therefore, an urgent need to make diagnostic and specific therapeutic units available in public health services for this population group, which is still socially active.

Audiology for the elderly is a recent development in public health,^37^ particularly in developing countries such as Brazil. The current demographic distribution is also fairly recent,^38^ revealing a higher number of youthful elderly people that are socially active and who wish to enjoy a vast social agenda; in fact, this group has attained increased social visibility.

Another important finding is that there was a higher prevalence of mild hearing loss in all groups with hearing loss. Such information points to a need for preventive public health measures against further auditory loss. More effective policies than those currently available are needed to provide hearing aids.

A further measure would be to offer specific auditory retraining programs for the elderly that present hearing loss, which could increase their quality of life. Such programs would foster adaptation and daily use of hearing aids and could increase social interactions for this age group. Auditory retraining, language and communication techniques substantially facilitate the communication process, which may be altered even if there is mild hearing loss.

We believe that it is important to record the prevalence of hearing loss in subjects aged 65 years or more, based on a population study in the city of Rio de Janeiro. We hope to involve as many professionals and other people as possible by providing information and guidance about the social and health consequences and implications of hearing loss in the elderly.
